# Rare genetic heterogeneity within single tumor discovered for the first time in colorectal liver metastases after liver resection

**DOI:** 10.18632/oncotarget.25119

**Published:** 2018-04-24

**Authors:** Mylène Sebagh, Nelly Bosselut, Alexandre Dos Santos, Marc-Antoine Allard, Aldrick Ruiz, Raphaël Saffroy, Daniel Cherqui, Eric Vibert, Denis Castaing, René Adam, Antonio Sa Cunha, Antoinette Lemoine

**Affiliations:** ^1^ AP-HP Hôpital Paul Brousse, Laboratoire d’Anatomie Pathologique, Villejuif, France; ^2^ Inserm, Unité 1193, Université Paris-Saclay, Villejuif, France; ^3^ Univ Paris-Sud, UMR-S 1193, Université Paris-Saclay, Villejuif, France; ^4^ DHU Hepatinov, Villejuif, France; ^5^ AP-HP Hôpital Paul Brousse, Département d’Oncogénétique, Villejuif, France; ^6^ AP-HP Hôpital Paul-Brousse, Centre Hépato-Biliaire, Villejuif, France; ^7^ Inserm, Unité 935, Université Paris-Saclay, Villejuif, France; ^8^ University Medical Center Utrecht, Department of Surgery, Utrecht, The Netherlands

**Keywords:** colorectal liver metastases, intratumoral genetic heterogeneity, pathological response, mutations

## Abstract

Effective individualized treatment of patients with colorectal liver metastases (CLM) requires tumor genotyping, usually based on the analysis of one single sample per patient. Therapy failure may partially be explained by sampling errors and/or intratumoral genetic heterogeneity. We aimed to demonstrate intratumoral genetic heterogeneity in CLM and enable pathologists to select tumor tissue for genotyping. All the tumors of 86 patients who underwent liver resection for a single CLM were reviewed. Of the 86 patients, 66 patients received chemotherapy and 20 patients did not receive chemotherapy before liver resection. All the tumor areas sampled were analyzed for *KRAS*, *BRAF*, *PIK3CA*, and *NRAS* mutations. The mutational status was tested in 74 cases, 7 cases had no tumoral cells due to complete responses and 5 blocks were unavailable. Of the 59/74 CLM with > 1 sample, 56 showed the same mutational status between the samples. The remaining 3 cases (5% of all cases) showed genetic heterogeneity for *KRAS* in 2 and *BRAF* in 1 patient. Genetic heterogeneity correlated with lower rate of viable tumor cells (p=0.009) and higher rate of mucin pools (p=0.013). We demonstrate for the first time the existence of genetic intratumoral heterogeneity in 5% of CLM. In routine practice, this low incidence does not require the genotyping of additional tumor samples. The correlation between the genetic heterogeneity and some histological components of the CLM should be verified by further *in situ* mutation assay.

## INTRODUCTION

During the last three decades, there has been evidence of a dramatic improvement in outcomes of patients with colorectal liver metastases (CLM), due to a multidisciplinary approach and advances in genetic profiling and mutational analysis. An adjunct to improved surgical techniques and systemic chemotherapy is the use of local therapy, radiological interventions, and the use of targeted agents.

The treatment of patients with CLM is now individualized and both based on pattern and extent of the metastatic disease and tumor genetics. Tumor genotyping is become standard practice for CLM and clinicians often have information on the mutational status of oncogenes, including the *KRAS*, *BRAF*, *PIK3CA*, and *NRAS* oncogenes. In addition to the prediction of response to targeted agents, these mutations may affect the metastatic behavior of tumors and patterns of metastatic spread [1].

Usually only one single sample is analyzed per patient. However, no general recommendation exists as to which tumor sample should preferentially be tested. Specific questions refer to the choice of the tumor tissue: 1) primary tumor and/or metastases. Many studies compared the mutational status between primary colorectal tumor and liver metastases. The majority of them found a concordance supporting that *KRAS* mutation is an early event in CRC tumorigenesis [2–8]; 2) which CLM to choose in patients with multiple CLM. By analyzing all the CLM in a given patient, we recently assessed the existence of intermetastatic heterogeneity between the CLM on a pathological basis [9]. Furthermore, genetic heterogeneity was present in 30% of these patients with pathological heterogeneity; 3) finally, which part of the chosen CLM. This latter question raises the issue of possible genetic intrametastatic heterogeneity, which prompted the current study. This could subsequently help pathologists in the selection of the block for the genotyping.

## RESULTS

### Chemotherapy (CT) group

#### Clinical patient characteristics

The CT group included 45 men and 21 women with a median age of 64 years (34-84 years). The primary tumour was located in the colon and the rectum in 54 (82%) and 12 patients (18%), respectively. The primary tumour was graded T1/T2 in 10 patients (15%), T3/T4 in 51 patients (77%) and Tx in 5 patients (7%). CLM were synchronous to the primary tumor in 39 (59%) patients. Thirty-nine patients (33%) had regional node-positive primary colorectal carcinoma.

Of the study population, 29 patients received oxaliplatin-based chemotherapy, 35 patients received irinotecan-based chemotherapy, 19 received bevacizumab, and 10 patients received cetuximab. The median number of chemotherapy cycles was 6 (range, 3-25).

#### Pathological features

The median tumor size was 3 cm (range, 0.3 to 17 cm). A total of 178 samples with a mean of 2.7 and a median number of 2 were reviewed.

Seven patients had a complete response. The pathological response was as follows: According to the method by Blazer et al. [10], the mean and median rates of residual tumor cells were 34.9% and 40% (range, 0-90%), respectively. According to the method by Sebagh et al. [11], the mean and median scores were 1.23 and 0.78 cm-residual tumor (range, 0-7.6).

#### Gene mutation status(Figure 1)

The mutational status was not tested necessarily in the 7 patients with complete response and in 4 other patients with unavailable blocks. A total of 167 samples (mean= 3; median= 3) in the remaining 55 patients were tested for the genetic profiling. Of them, 36 patients did not exhibit mutations and 19 patients had one or more mutations: 16 patients had *KRAS* mutations (on exon 2: 14 patients and on exon 3-4: 2 patients), 1 *NRAS* mutation, 2 *BRAF* mutation and 4 *PIK3CA* mutations, associated with *KRAS* mutations in all of them (on exon 2: 3 patients and on exon 3-4: 1 patient).

Of them, 44 patients underwent more than 1 sample per CLM. The same mutational status in all the samples was observed in 41 patients (93.2%): All the samples were wild-type in 27 patients and mutated in 14 patients. When mutated, the same type of mutation was observed. In the remaining 3 patients (6.8%), the mutational status differed between the samples: In 2 patients, one of their 3 and 4 samples, respectively, harbored a *KRAS* mutation (on exon 2) while the other samples were wild-type. In the third patient, one of his 3 samples harbored a *BRAF* mutation while his 2 other samples were wild-type (Figure 2).

**Figure 1:**
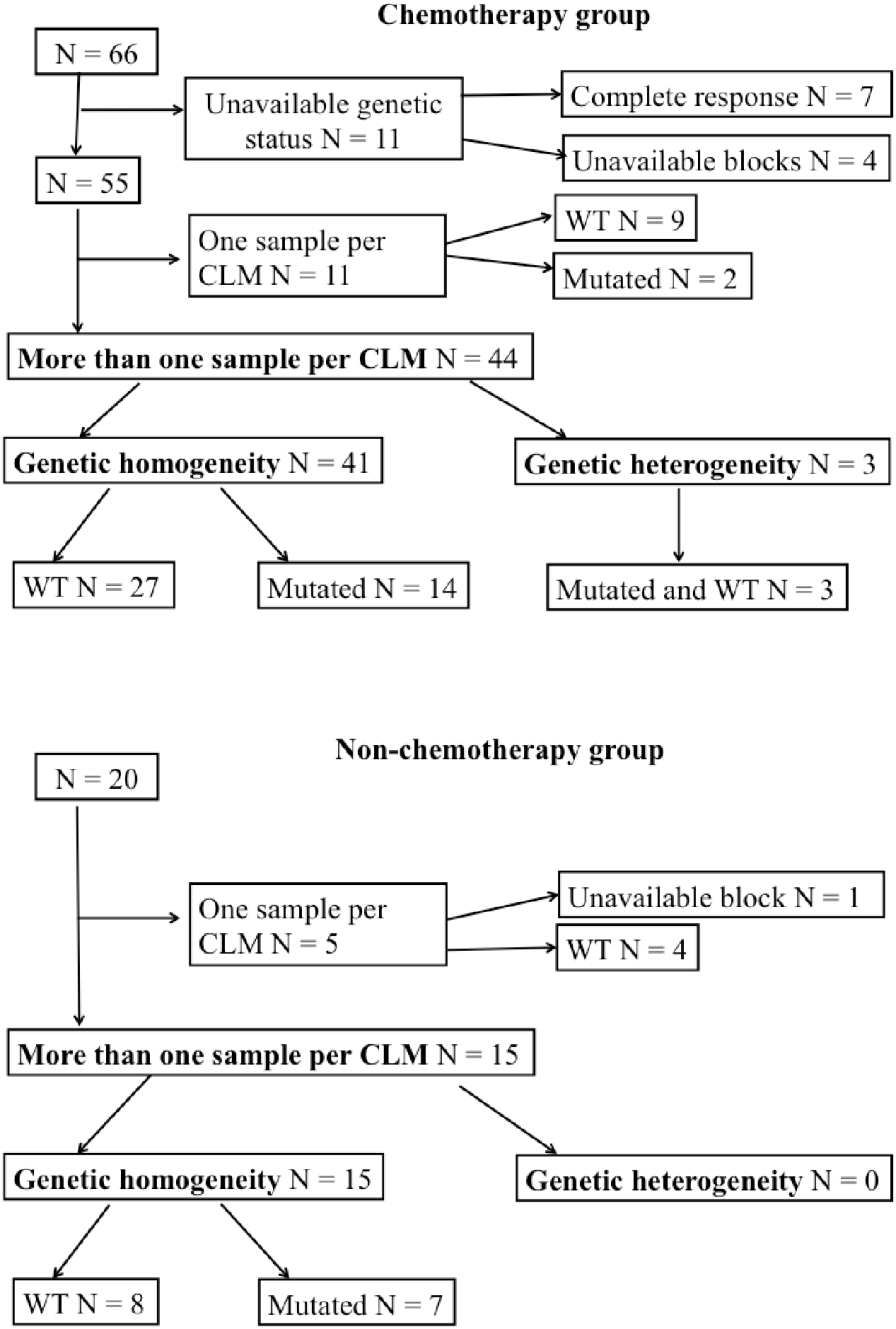
Gene somatic profile in the chemotherapy and non-chemotherapy groups, respectively

**Figure 2:**
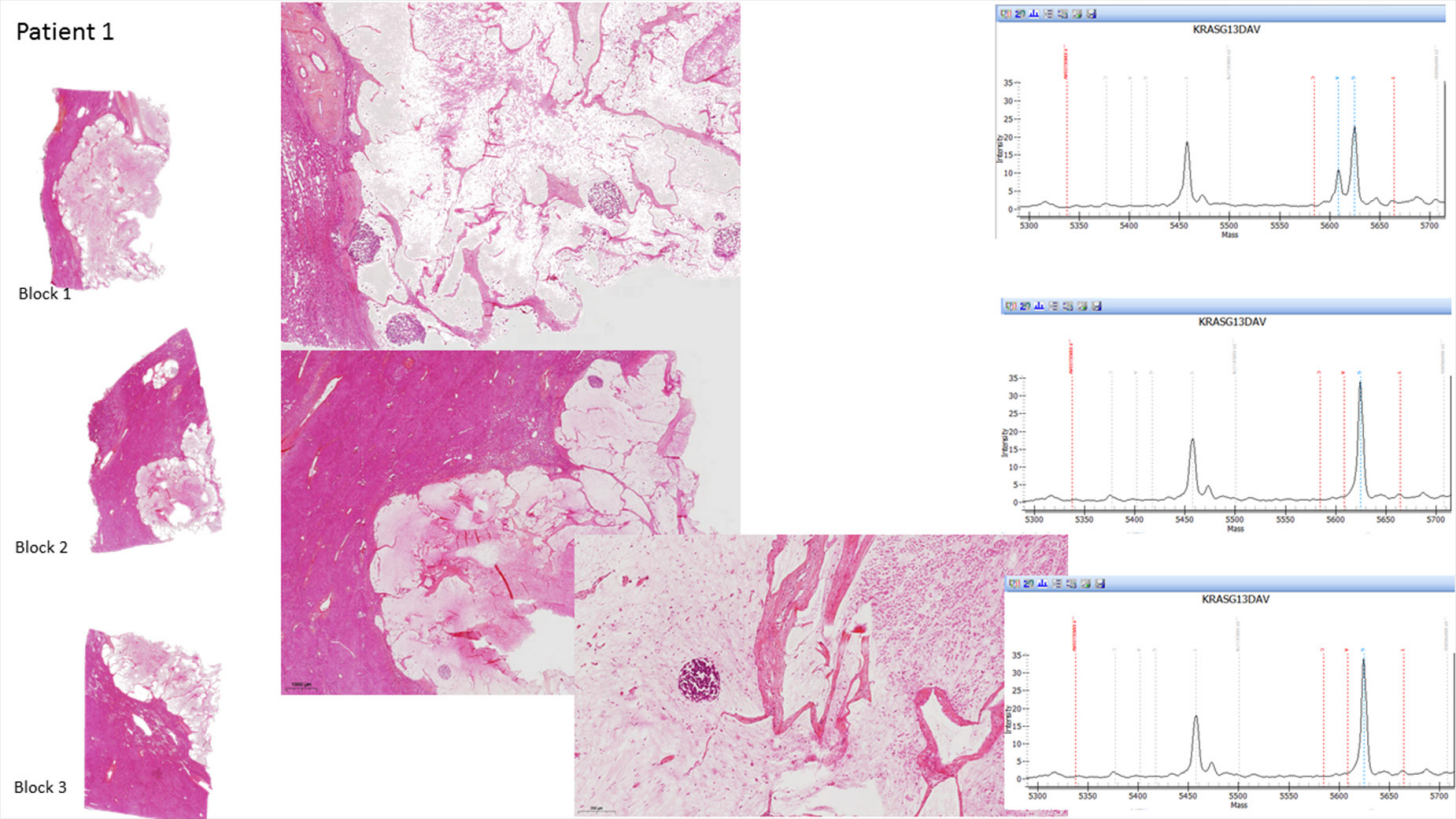

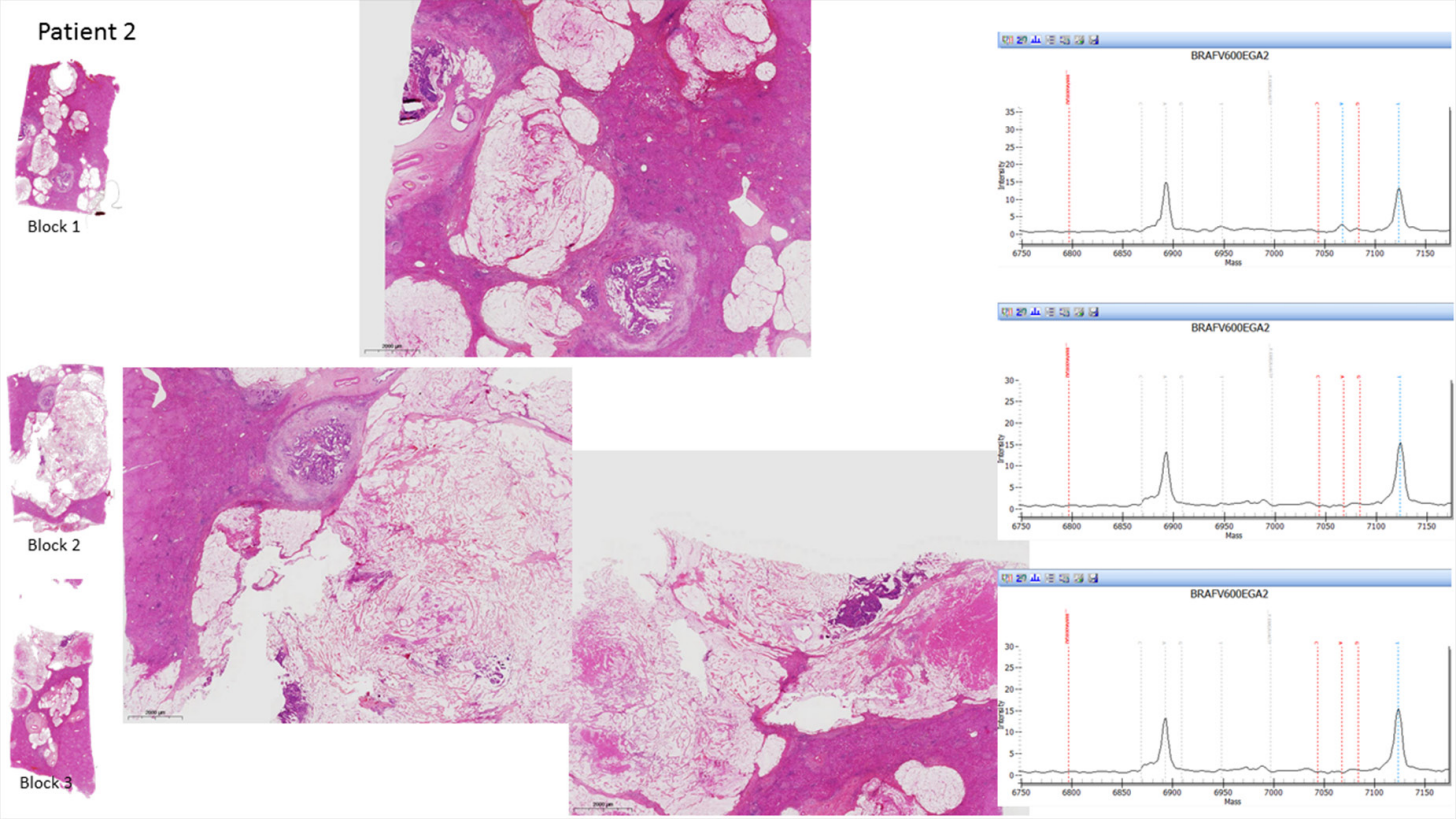

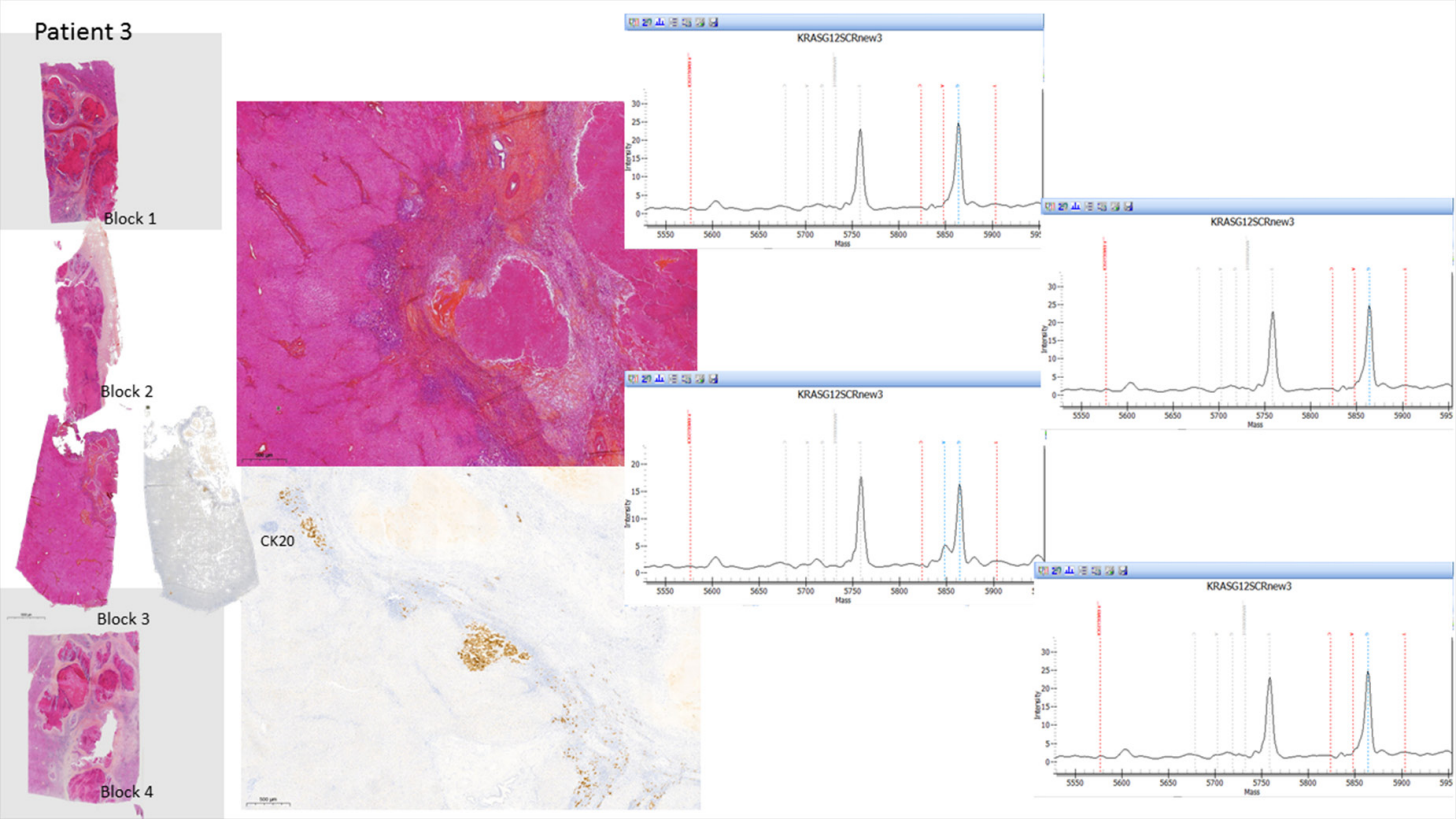
Histopathological examination of the H&E-stained sections for each tumor area sampled and the corresponding molecular data in the 3 CLM with genetic heterogeneity

### Non-chemotherapy (non-CT) group

Over the study period, 20 patients (14 men and 6 women, with a mean age of 67 years) were resected for a single nodule without preoperative chemotherapy.

The primary tumour was located in the colon and the rectum in 14 (30%) and 6 patients (70%), respectively. The primary tumour was graded T1/T2 in 6 patients, T3/T4 in 13 patients and Tx in 1 patients. CLM were synchronous to the primary tumor in 6 (30%) patients. Seven patients had regional node-positive primary colorectal carcinoma. The median tumor size was 2.6 cm (range: 0.5 to 7.5 cm).

In the non-CT group, 15 had more than one sample per CLM (median=2, range 2 to 3). All of them showed genetic homogeneity between the samples: 7 were all mutated (4 *KRAS* on exon 2, 1 *KRAS* on exon 3-4, 1 *PIK3CA* and 1 *NRAS*) and 8 were all wild-type (Figure 1).

### Relationship between various clinical and pathological factors, and intratumoral genetic heterogeneity in the overall population (Table 1)

Regarding the clinical variables, the presence of genetic heterogeneity significantly correlated with female gender (p=0.025) and primary tumor stage (T1/2 versus T3/4), but not with age, primary tumor location, timing of metastases (synchronous versus metachronous), use of preoperative chemotherapy, number of preoperative chemotherapy cycles, or use of targeted agent.

**Figure 3:**
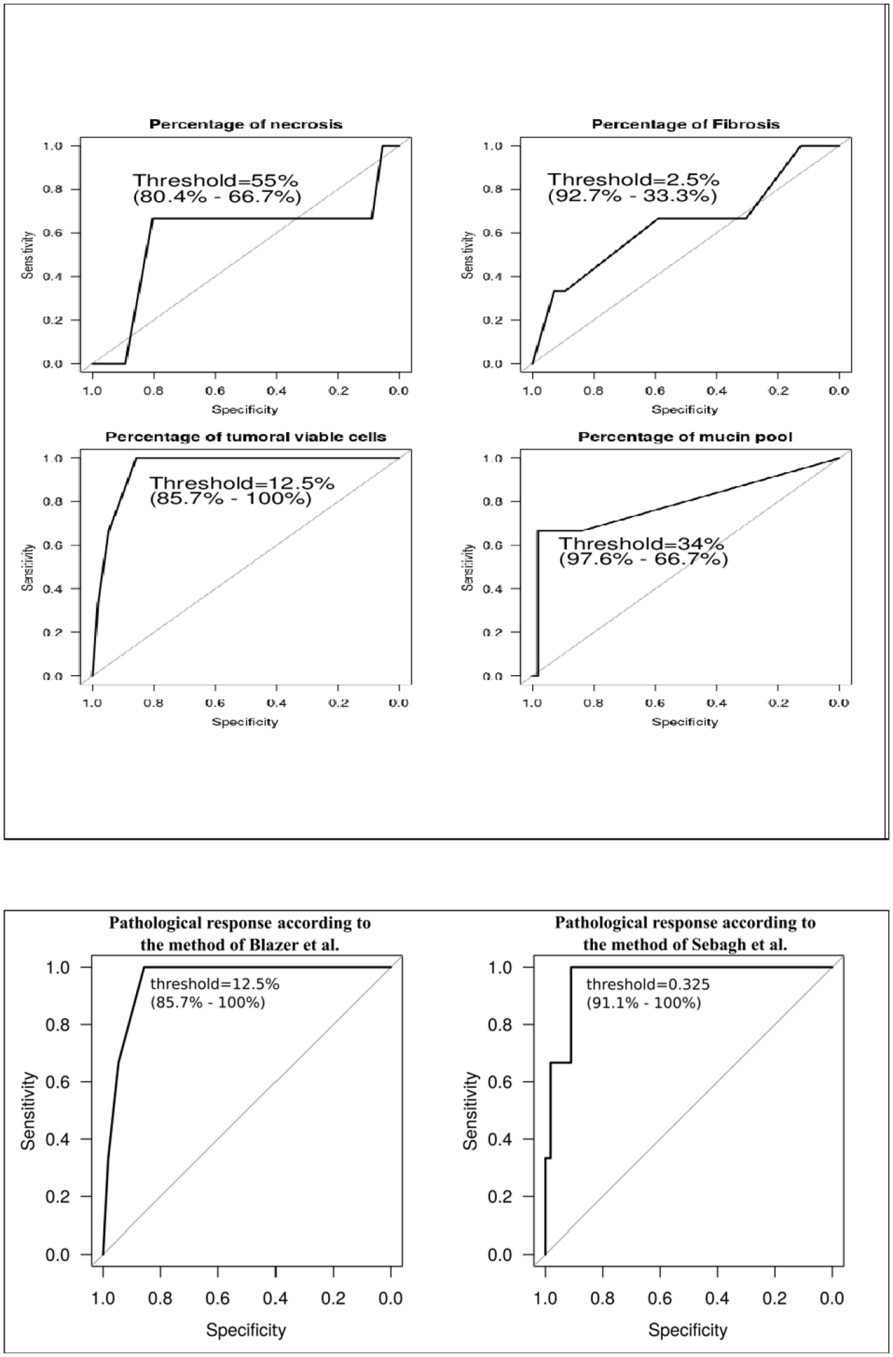
Relationship between the presence of intratumoral genetic heterogeneity and pathological variables in the overall population The better threshold was 34% (specificity of 97.6%; sensitivity of 66.7%) for the rate of mucin pools, 12.5% for the rate of viable tumor cells as well as for the pathological response according to the method by Blazer (specificity of 85.7%; sensitivity of 100%) and 0.3 cm-residual tumor for the pathological response according to the method by Sebagh (specificity of 91.1%; sensitivity of 100%).

Regarding the pathological variables, the presence of genetic heterogeneity was not correlated with tumor size, number of tumor areas sampled or the ratio number of tumor areas sampled/tumor size. The presence of genetic heterogeneity significantly correlated with higher rates of mucin pools (p=0.013) and lower rate of viable tumor cells (p=0.009), but not with coagulative necrosis and fibrosis rates. Genetic heterogeneity correlated with the pathological response assessed by both methods (p=0.009 and 0.007, respectively). Multivariate analysis was not performed considering the small group with genetic heterogeneity. Figure 3 identified that the better threshold was 34% (specificity of 97.6%; sensitivity of 66.7%) for the rate of mucin pools, 12.5% for the rate of viable tumor cells as well as for the pathological response according to the method by Blazer (specificity of 85.7%; sensitivity of 100%) and 0.325 cm-residual tumor for the pathological response according to the method by Sebagh (specificity of 91.1%; sensitivity of 100%).

**Table 1:** Univariate analysis of clinical and pathological variables associated with genetic heterogeneity

	Genetic heterogeneity		p-value
	yes	no	
**Age** (y)	59 (54-75)	64 (35-87)	0.806
**Sex** (M/F)	0/3	41/15	**0.025**
**Primary tumor location** (colon/rectum)	3/0	41/14	1
**Primary tumor stage**			
T1/T2	0	8	**0.004**
T3/T4	3	45	1
N/A	0	6	1
**Timing of metastases** (synchronous/ metachronous)	2/1	22/34	0.556
**Preoperative chemotherapy** (yes/no) **Number of preoperative chemotherapy cycles**	3/0 5 (5-12)	41/15 5 (0-13)	0.563 0.365
**Targeted agents**			
Bevacizumab	2	15	0.545
Cetuximab	1	5	0.374
one of them	3	20	0.0545
**Tumor size** (cm)	3 (2.9-3)	4.4 (0.8-17)	0.291
**Number of samples**	3 (3-4)	3 (2-7)	0.414
**Number of samples per cm**	1.034 (1-1.33)	0.903 (0.294-3.33)	0.390
**Components of the CLM**			
Viable tumor cell rate (%)	5 (2-10)	39 (2-90)	**0.009**
Fibrosis rate (%)	10 (0-30)	21 (0-80)	0.409
Necrosis rate (%)	60 (5-60)	35 (0-95)	0.621
Mucin pool rate (%)	38 (0-80)	5 (0-98)	**0.013**
**Pathological response**			
Method by Blazer et al	5 (2-10)	39 (2-90)	**0.009**
Method by Sebagh et al	0.15 (0.0580-0.3)	1.58 (0.058-7.6)	**0.007**

## DISCUSSION

In patients with wild-type *KRAS, BRAF* and *PIK3CA*, the response was not more than 40% to 60%. Conversely, patients with tumors apparently lacking EGFR expression responded to antibody therapy in up 25% of the cases. This lack of correlation could at least partially be explained by sampling errors and inter- and intratumoral heterogeneity [12]. This study focused onthe presence of genetic intratumoral heterogeneity in patients resected for a single CLM, by evaluating the target genes by molecular analyses from all the tumor areas sampled. This provided a recommendation in the selection of blocks for the genotyping according to the histological components of CLM.

Tumor genotyping is become standard practice for CLM and clinicians often have information on the mutational status of oncogenes, including the *KRAS*, *BRAF*, *PIK3CA*, and *NRAS* oncogenes. Usually only one single sample is analyzed per patient. The choice of tumor tissue is a crucial step. Material from metastatic sites is not routinely collected, generally of poor quality and contains few tumor cells as a result of tumor necrosis or chemotherapy-induced changes. Among the different metastatic sites, lymph nodes are least suitable for diagnostic mutation analysis [4, 12]. To avoid necrosis, the genomic profiling should be preferentially evaluated in primary tumor biopsies before any treatment. However, small biopsies may contain insufficient tumor material. Additionally, biopsies cannot be representative of the whole tumor [13].

Intratumoral heterogeneity of *KRAS* mutations was extensively reported in primary colorectal carcinomas. In terms of frequency, intratumoral heterogeneity of *KRAS* was observed in 35% to 47% ^5–7,11^). In the series of 78 cases by Al-mulla et al. [3], 9 of 26 (34%) primaries with *KRAS* mutations also contained areas of carcinoma with only the wild-type gene, but the number of samples was not given. In the series by Giaretti et al. [6], intratumor *KRAS* heterogeneity was present in 3 of 9 mutated cases (33%) or 3 of a total of 19 adenocarcinomas (16%), by examining a multiple set of samples from both superficial and deep primary tumor sectors (from 6 to 11 per resection). Losi et al. [5] showed that intratumoral *KRAS* heterogeneity was detected in 9 of the 14 mutated cases (64%) or 9 of a total 25 adenocarcinomas (36%). Baldus et al. [12] compared two samples from tumor centers and invasion fronts in a large series of 100 cases. They showed intratumoral heterogeneity of *KRAS* in 8% of all cases and 20% of tumors with mutated *KRAS*. The rate of heterogeneity for *BRAF* and *PIK3C* were 1% and 5% of primary tumors, respectively.

The discrepancies regarding the frequency of intratumoral heterogeneity could be explained by the low number of cases investigated in most of these studies, but also regional genetic variation, number of tumor areas sampled and stage of the disease. Interestingly, Baldus et al. could successfully map the regional genetic variation [12]. They observed a higher rate of *KRAS* mutation in the tumor center compared with the front invasion, suggesting that tumor samples should be preferably taken from the tumor center, while no information was given if the profiling differed between both superficial and deep primary tumor sectors [5, 6]. The *KRAS* mutation could finally also be detected in the primary tumor, when additional tumor samples were tested [12]. Intratumoral heterogeneity for *KRAS* mutations also varied during progression of the disease [5, 12, 14]. It was significantly reduced from the early to the advanced stages from 60 to 20% [5]: in 10 early primaries (T1 or T2), *KRAS* mutation was detected in 6 cases (60%) and all of them showed an intratumoral genetic heterogeneity. In 15 advanced primaries (T3N1M0 or T4N1M0), *KRAS* mutation was detected in 8 tumors (53%) and 3 of these tumors were found to be heterogeneous. Fukunari et al. suggested that intratumoral heterogeneity could play a role in the enhancement of aggressive progression and the metastases of colorectal carcinomas. Liver metastasis occurred in 75% of the heterogeneous carcinomas, whereas hepatic metastasis occurred in only 12.5% of homogeneous carcinomas [14].

In accordance with this evolutionary concept, we could expect a low rate of intratumoral heterogeneity in CLM. To our knowledge, only 2 studies focused on this issue. In the series of 20 metastatic cases by Losi et al. [5], a quasi-absence of intratumoral genetic heterogeneity was observed for *KRAS* in distant metastasis: one case in a peritoneal metastasis and any of the 9 CLM. In the series by Fukunari et al. [14], no intratumoral heterogeneity was observed in the 7 CLM. Our current series identified for the first time intratumoral heterogeneity in 5% of all tumors. Intratumoral heterogeneity was present in 2 patients for *KRAS* and one patient for *BRAF*. All of them received chemotherapy and biotherapy before surgery. No significant correlation between genetic heterogeneity and the use of neither preoperative chemotherapy nor biotherapy was observed in the totality of included patients. An interesting aspect to consider is the timing of the genetic changes. Our findings were detected after patients had undergone preoperative chemotherapy and raise the question whether the genetic heterogeneity is an effect of treatment or tumor biology. Extrapolating from published analysis of primary tumor genetic makeup and from our statistical data, one might assume the latter [3, 5, 6, 12].

The main strengths of our study are the inclusion of a higher number of patients investigated, the histologic reviewing by a single pathologist, and the molecular analyses of the target genes evaluated in all the tumor areas sampled. However, it is illusory to have tested the whole tumor, which is the main limitation of this study and all others. The number of tumor samples at the time of macroscopic management of specimens usually depends on the tumor size. Small CLM is entirely sampled, while bigger CLM is undergone one sample per cm along the biggest dimension [15]. This recommendation may provide a potential bias between small and bigger CLM. In our series, genetic heterogeneity did not correlate with the tumor size, the number of samples or the ratio number of samples per tumor size. Another limitation of this study was the lack of microdissection. Owing to the low proportion and dispersion of tumor cells in the metastases, a microdissection of these tissues is often unfeasible in routine practice [5]. Our Oncogenetic Department performed mutation analyses on formalin-fixed paraffin-embedded tumors in the daily routine practice and adapted the technologies to increase the performance of the molecular tests with high sensitivity below 1%. To overcome false-negative samples, tumor cells have been enriched by manual macrodissection of normal parenchymal cells, and large necrotic and fibrotic areas within the CLM. Tumor samples harboring at least 10% tumoral cells were analyzed.

In routine practice, the genotyping from all tumor areas sampled of all CLM is unfeasible because such strategy is time and money consuming. We recently assessed the existence of pathological intermetastatic heterogeneity of more than 50% in 20% of patients with multiple CLM [9]. Genetic heterogeneity was present in 30% of these patients with pathological heterogeneity. The mutation was harbored by the less florid CLM in 75% of cases with genetic heterogeneity. We proposed to test additional CLM in case of wild-type results in the particular subpopulation of patients with pathological heterogeneity. We here identify for the first time the existence of genetic intratumoral heterogeneity in CLM. However, its incidence is low. This finding does not involve a change in the routine management, especially in the number of tissue samples to genotype: One enriched sample from a given CLM is likely sufficient in the large majority of cases. In case of therapy failure in wild-type patients, we should propose to genotype additional samples, especially from the CLM containing few tumor cells and/or abundant mucin pools, as we showed that genetic heterogeneity significantly correlated with the histological components of CLM. Although significant, this statistical correlation has to be taken into account with caution with respect to the low incidence of heterogeneity. It has to be verified in prospective studies, especially using *in situ* mutation detection, which directly combines histological examination and molecular diagnostics. For the first time, Grundberg et al. developed an RNA-based *in situ* mutation assay in colon and lung cancers [16]. Their *in situ* results for KRAS mutations were reliable and concordant with pyrosequencing of DNA extracts from the same tissues. The authors were able to apply this technique on frozen tissues, routinely collected formalin-fixed paraffin-embedded tissues and even cytology preparations. The assay was adapted for a multiplex detection of a set of relevant cancer targets. The sole limitation recognized by the authors is the number of available fluorophores to separate from each other. This *in situ* assay holds great promise as a tool to better understand the intratumoral heterogeneity. From a practical point of view, the visualization of a potential genetic heterogeneity requires the application of the *in situ* assay in a multiplex fashion and on all the tumor areas sampled. The adaptation of the *in situ* protocol for automation should facilitate implementation of the assay for routine use.

In conclusion, this study provides for the first time the existence of genetic intratumoral heterogeneity in 5% of CLM. This low incidence supports the evolutionary concept of its decrease during the disease progression. In routine practice, because of this low incidence, the genotyping of additional tumor samples is not required in most cases. Genetic intratumoral heterogeneity seems to correlate with particular histological components of CLM. This finding should be confirmed by further studies. In cancer research, *in situ* mutation assay should be a promise tool to vizualize and elucidate the genetic intratumoral heterogeneity.

## PATIENTS AND METHODS

### Patients and tumor samples

The population consisted of the patients who underwent elective liver resection for CLM at Paul Brousse Hospital (Villejuif, France) between 2004 and 2011, and in whom the tissue material was available for pathologic review. Because we recently showed that pathological heterogeneity correlated with a greater number of CLM [9], we chose to include patients resected for a single CLM according to 2 groups: The first group (Chemotherapy group) consisted of patients operated after at least 3 cycles of preoperative chemotherapy with no more than two lines in association; the second group (Non-chemotherapy group) consisted of those who did not undergo preoperative chemotherapy.

At the time of macroscopic management, CLM < 2 cm were entirely sampled in one or 2 blocks and bigger lesions were extensively sampled from the center to the periphery (Supplementary Figure 1). Formalin-fixed paraffin embedded tissue blocks were cut at 4 μm thickness and stained with haematoxylin and eosin. A single pathologist blinded to clinical information has reviewed all stained sections. The percentages of area with remaining viable tumour cells, coagulative necrosis, mucin pools and fibrosis in relation to the total area of the CLM were evaluated in each CLM. The method by Blazer (percentage of residual tumor cells) [10] and by Sebagh (percentage of residual tumor cells x CLM size in centimeter) [11] were used for the assessment of the pathological response.

### Tumour DNA preparation and gene mutation profiling

Following the assessment of the percentage of tumor cells in relation with the sample area (including non tumoral liver and stroma of the tumor), the block was subsequently cut at 30 μm and macrodissected if containing less than 10% of tumor cells (Supplementary Figure 1). DNA extraction was performed using the QIAmp DNA Mini kit (Qiagen, Courtaboeuf, France) according to the manufacturer instructions. For the purpose of the study, DNA extraction was performed within all the tumor areas sampled from a given CLM. All the samples were retrospectively and completely tested for the relevant genes in primary colorectal tumor and CLM (*i.e., KRAS, NRAS, BRAF* and *PIK3CA*). Somatic gene mutations were detected using the MassARRAY iPLEX platform (Sequenom, San Diego, US), which involves a three-step process consisting of the initial PCR reaction, inactivation of unincorporated nucleotides by shrimp alkaline phosphatase and a single-base primer extension. Then, the products are nano-dispensed onto a matrix-loaded silicon chip (SpectroChipII, Sequenom, San Diego, US) and finally, the mutations are detected by MALDI–TOF (matrix-assisted laser desorption-ionization–time of flight) mass spectrometry. The experimental sensitivity of the assay was estimated to be below 1% for each gene mutation.

### Statistical analysis

Continuous data were expressed as median (range) and/or mean (standard deviation) whereas categorical data were expressed as percentage. Continuous data were compared using Wilcoxon rank sum test and categorical data were compared using Fisher's exact test. Univariate and multivariate analysis were used to examine the relationship between various clinical and pathological factors, and genetic heterogeneity (as present or absent). In order to evaluate biomarker performance, ROC curves were performed on continuous variables using pROC package for R.

## SUPPLEMENTARY MATERIALS FIGURE



## References

[1] Lipsyc M, Yaeger R (2015). Impact of somatic mutations on patterns of metastasis in colorectal cancer. J Gastrointest Oncol.

[2] Knijn N, Mekenkamp LJ, Klomp M, Vink-Borger ME, Tol J, Teerenstra S, Meijer JW, Tebar M, Riemersma S, van Krieken JH, Punt CJ, Nagtegaal ID (2011). KRAS mutation analysis: a comparison between primary tumours and matched liver metastases in 305 colorectal cancer patients. Br J Cancer.

[3] Al-Mulla F, Going JJ, Sowden ET, Winter A, Pickford IR, Birnie GD (1998). Heterogeneity of mutant versus wild-type Ki-ras in primary and metastatic colorectal carcinomas, and association of codon-12 valine with early mortality. J Pathol.

[4] Vakiani E, Janakiraman M, Shen R, Sinha R, Zeng Z, Shia J, Cercek A, Kemeny N, D'Angelica M, Viale A, Heguy A, Paty P, Chan TA (2012). Comparative genomic analysis of primary versus metastatic colorectal carcinomas. J Clin Oncol.

[5] Losi L, Baisse B, Bouzourene H, Benhattar J (2005). Evolution of intratumoral genetic heterogeneity during colorectal cancer progression. Carcinogenesis.

[6] Giaretti W, Monaco R, Pujic N, Rapallo A, Nigro S, Geido E (1996). Intratumor heterogeneity of K-ras2 mutations in colorectal adenocarcinomas: association with degree of DNA aneuploidy. Am J Pathol.

[7] Etienne-Grimaldi MC, Formento JL, Francoual M, François E, Formento P, Renée N, Laurent-Puig P, Chazal M, Benchimol D, Delpero JR, Letoublon C, Pezet D, Seitz JF (2008). K-Ras mutations and treatment outcome in colorectal cancer patients receiving exclusive fluoropyrimidine therapy. Clin Cancer Res.

[8] Santini D, Loupakis F, Vincenzi B, Floriani I, Stasi I, Canestrari E, Rulli E, Maltese PE, Andreoni F, Masi G, Graziano F, Baldi GG, Salvatore L (2008). High concordance of KRAS status between primary colorectal tumors and related metastatic sites: implications for clinical practice. Oncologist.

[9] Sebagh M, Allard MA, Bosselut N, Dao M, Vibert E, Lewin M, Lemoine A, Cherqui D, Adam R, Sa Cunha A (2016). Evidence of intermetastatic heterogeneity for pathological response and genetic mutations within colorectal liver metastases following preoperative chemotherapy. Oncotarget.

[10] Blazer DG, Kishi Y, Maru DM, Kopetz S, Chun YS, Overman MJ, Fogelman D, Eng C, Chang DZ, Wang H, Zorzi D, Ribero D, Ellis LM (2008). Pathologic response to preoperative chemotherapy: a new outcome e nd point after resection of hepatic colorectal metastases. J Clin Oncol.

[11] Sebagh M, Allard MA, Cunha AS, Ruiz A, Araujo R, Lemoine A, Paule B, Delvart V, Cherqui D, Vibert E, Adam R (2014). A proposed new method for assessing the pathological response to chemotherapy in resected colorectal liver metastases. Br J Cancer.

[12] Baldus SE, Schaefer KL, Engers R, Hartleb D, Stoecklein NH, Gabbert HE (2010). Prevalence and heterogeneity of KRAS, BRAF, and PIK3CA mutations in primary colorectal adenocarcinomas and their corresponding metastases. Clin Cancer Res.

[13] Bettoni F, Masotti C, Habr-Gama A, Correa BR, Gama-Rodrigues J, Vianna MR, Vailati BB, Sao Juliao GP, Fernandez LM, Galante PA, Camargo AA, Perez RO (2017). Intratumoral Genetic Heterogeneity in Rectal Cancer: Are Single Biopsies representative of the entirety of the tumor?. Ann Surg.

[14] Fukunari H, Iwama T, Sugihara K, Miyaki M (2003). Intratumoral heterogeneity of genetic changes in primary colorectal carcinomas with metastasis. Surgery Today.

[15] Rubbia-Brandt L, Giostra E, Brezault C, Roth AD, Andres A, Audard V, Sartoretti P, Dousset B, Majno PE, Soubrane O, Chaussade S, Mentha G, Terris B (2007). Importance of histological tumor response assessment in predicting the outcome in patients with colorectal liver metastases treated with neo-adjuvant chemotherapy followed by liver surgery. Ann Oncol.

[16] Grundberg I, Kiflemariam S, Mignardi M, Imgenberg-Kreuz J, Edlund K, Micke P, Sundström M, Sjöblom T, Botling J, Nilsson M (2013). *In situ* mutation detection and visualization of intratumor heterogeneity for cancer research and diagnostics. Oncotarget.

