# Supplementary material for: Rare genetic heterogeneity within single tumor discovered for the first time in colorectal liver metastases after liver resection
Source: Oncotarget. 2018 Apr 24;9(31):21921–9. doi: 10.18632/oncotarget.25119 (PMC5955166; doi:10.18632/oncotarget.25119)
Supplement: Supplementary file 1 [file oncotarget-09-21921-s001.pdf]

Rare genetic heterogeneity within single tumor discovered for the first time in colorectal liver metastases after liver resection

SUPPLEMENTARY MATERIALS

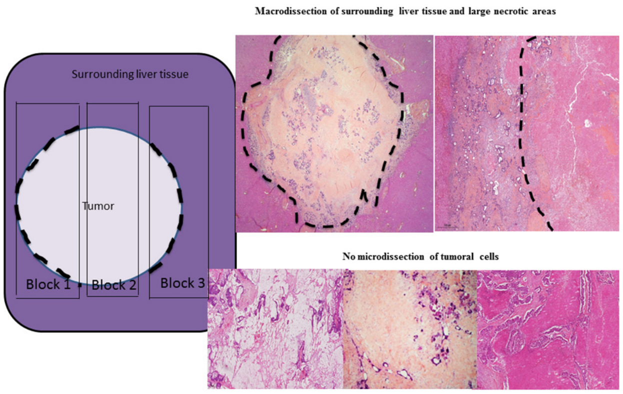

Supplementary Figure 1: Preparation of paraffin-embedded material for genetic analyses. Tumor cells have been enriched by manual macrodissection of normal parenchymal cells, and large necrotic and fibrotic areas within the CLM. Owing to the low proportion and dispersion of tumor cells in the metastases, a microdissection of these tissues was not performed.
